# Giant Gastroschisis with Complete Liver Herniation: A Case Report of Two Patients

**DOI:** 10.1155/2019/4136214

**Published:** 2019-01-15

**Authors:** Wendy Jo Svetanoff, Benjamin Zendejas, Farokh R. Demehri, Alex Cuenca, Bharath Nath, C. Jason Smithers

**Affiliations:** ^1^Department of General Surgery, Boston Children's Hospital, 300 Longwood Ave, Fegan 3, Boston MA 20115, USA; ^2^Department of Transplant Surgery, Massachusetts General Hospital, 165 Cambridge Street, Suite 301, Boston, MA 02114, USA

## Abstract

**Introduction:**

There are no reported survivors of gastroschisis with complete liver herniation. We describe a case report of two patients, one of whom survived.

**Case #1:**

The patient was born with gastroschisis and herniation of the entire liver. Along with silo placement, the abdominal fascia was attached to an external traction system for growth. Complete closure was achieved at 5 months. Due to pulmonary hypoplasia, high-frequency ventilation was required. The patient is doing well, on a home ventilator wean, at 20 months.

**Case #2:**

The patient was born prematurely with gastroschisis, total liver herniation, and a defect extending to the pericardium. A silo was attached to the fascia to provide growth of the abdominal cavity. The patient developed respiratory failure, diffuse anasarca, and renal failure. She died at 38 days of life.

**Discussion:**

We report the first survivor of gastroschisis with complete liver herniation, contrasting it with a death of a similar case. The associated pulmonary hypoplasia may require long-term ventilation, the inflammatory response can lead to anasarca, and renal injury can occur from acute-on-chronic compartment syndrome.

**Conclusion:**

External fascial traction systems can help induce growth of the abdominal wall, allowing closure of the challenging abdomen. While critical care management is complex, survival is possible.

## 1. Introduction

Abnormal development of the abdominal cavity can lead to congenital defects such as gastroschisis and omphalocele [1, 2]. The type and size of defect can be determined with prenatal ultrasound. Other measurements, such as the lung/thorax transverse ratio and observed/predicted total lung volume (O/E TLV) on fetal MRI, can also be determined to estimate the amount of pulmonary hypoplasia associated with large defects, allowing for appropriate prenatal counseling [3, 4].

Omphaloceles and gastroschisis present in a wide spectrum of severity, primarily dependent on the size of the defect, the degree of the herniated contents, and the resulting associated malformations. The size cutoff depicting a large defect is variable. Some suggest that a major omphalocele may have a defect of 5, 6, or even 8 cm, with or without liver herniation [5]. Others have argued that a “large” omphalocele defect is >4 cm and includes the liver, while gastroschisis is usually believed to be less than 4 cm [2]. As a result of this discrepancy, Campos et al. recommended using the degree of visceroabdominal disproportion to classify the defect, as patients with high visceroabdominal disproportion often cannot be closed primarily due to the risk of abdominal compartment syndrome [5].

Patients with visceroabdominal disproportion have an abnormally narrow thorax and pulmonary hypoplasia, which can lead to respiratory insufficiency. This combination of findings indicates a poor prognosis with greatly decreased survival compared to their counterparts [2, 4–7]. Neither the management nor the survival of an infant with giant gastroschisis and total liver herniation has been described previously. In this report, we describe two cases of giant gastroschisis with complete loss of abdominal domain and total herniation of the liver; one survived and one did not; from such contrast, we draw and reflect on the lessons learned from caring for these patients.

## 2. Case Presentations

### 2.1. Case #1: Prenatal Care

**Figure 1:**
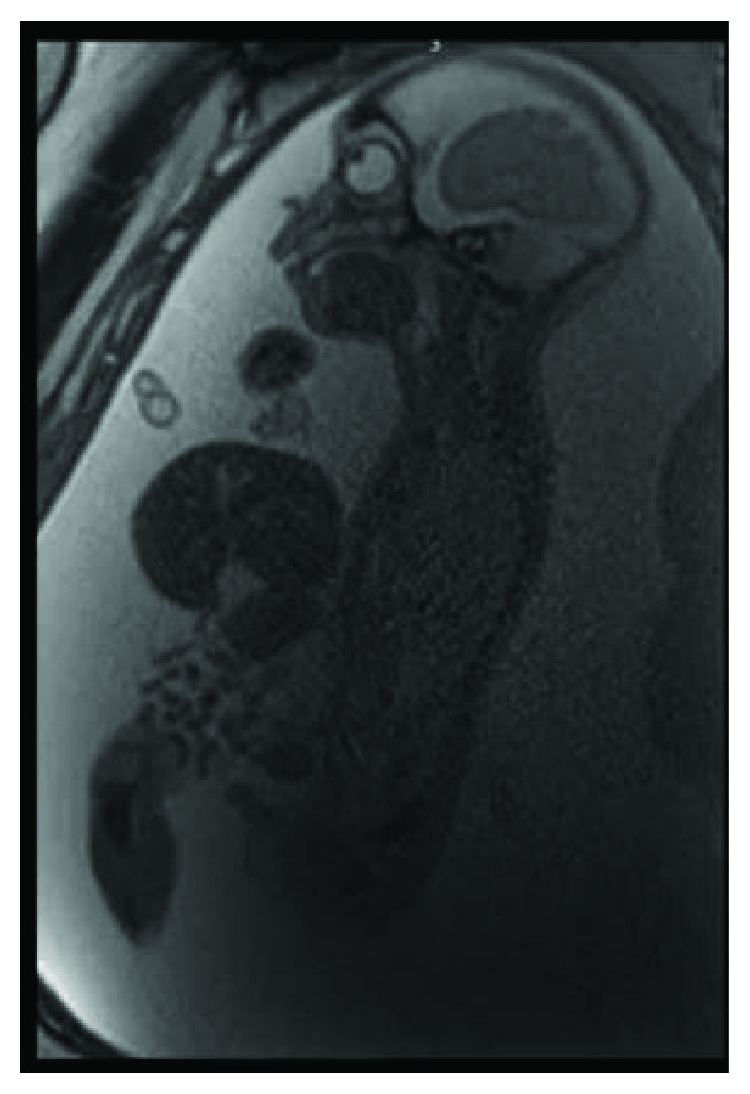
Prenatal imaging of patient #1. Prenatal imaging showed a large abdominal wall defect without evidence of a sac. Notice the small thoracic cavity, indicative of pulmonary hypoplasia.

The first case is a newborn male with a prenatal ultrasound and fetal MRI confirming an extensive anterior abdominal wall defect containing the small bowel, colon, all of the liver, and stomach without a definitive covering and pulmonary hypoplasia (Figure 1). After extensive discussion with the family, including prediction of a complex postnatal course and poor survival probability, the family elected to continue the pregnancy.

#### 2.1.1. Delivery and Initial Operation

**Figure 2:**
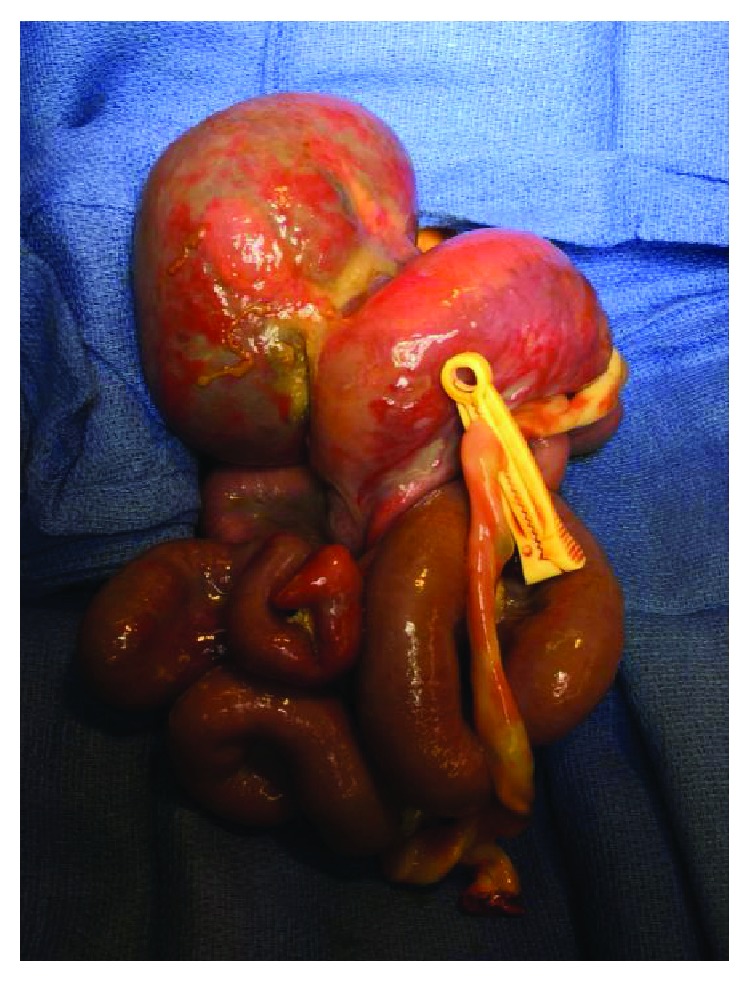
Gastroschisis contents at initial trip to the operating room. Upon birth, the patient was taken to the operating room for management of his massive gastroschisis. The liver has a rounded globular shape.

**Figure 3:**
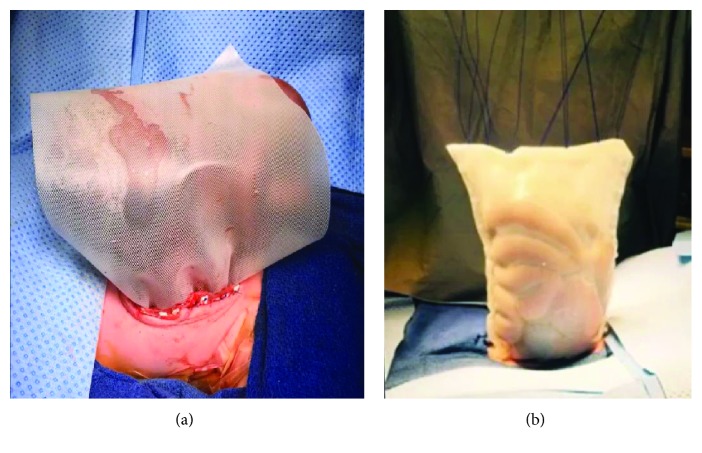
Placement of the silastic silo. Two silastic sheets were required to cover the entirety of the abdominal defect. Prolene suture was used to attach the two sheets together. Felt pledgets and Prolene sutures were used to attach the silastic silo to the abdominal fascia (a). (b) The completed silo.

**Figure 4:**
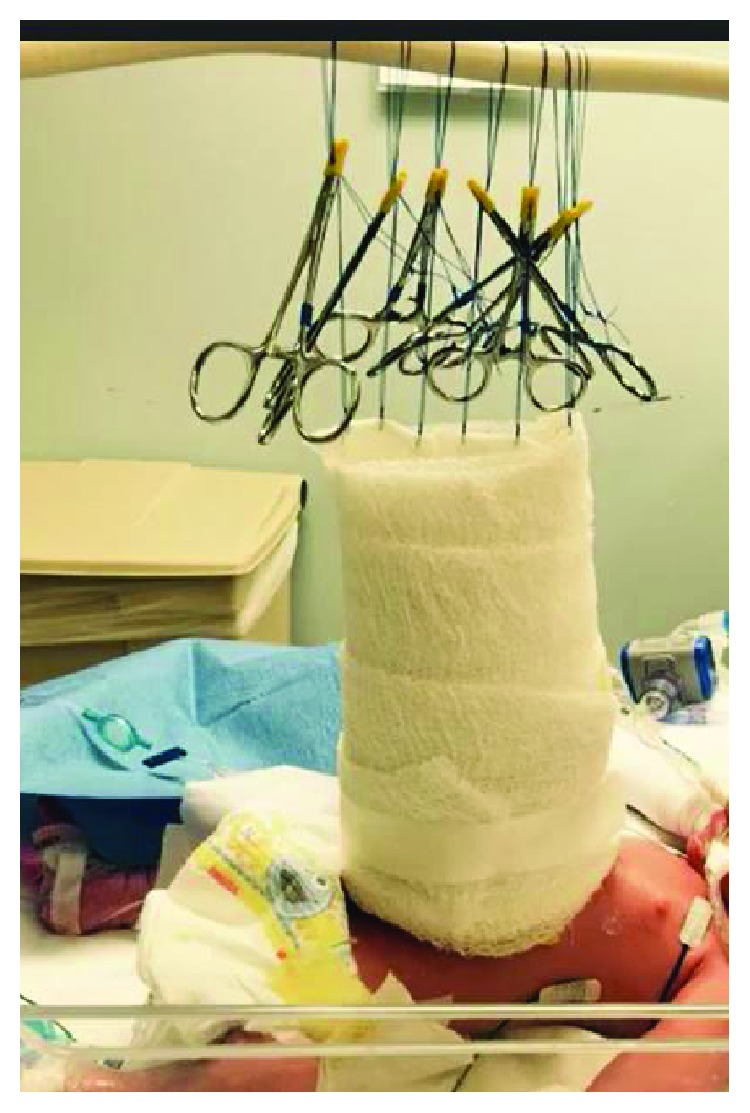
External traction system after dressing application was complete. Once the silo was placed under tension, betadine, petrolatum gauze, and dry gauze were used to keep the area sterile and prevent fluid loss.

The patient was born via scheduled caesarian section at 38-week gestation with an estimated birth weight of 3 kg. He required immediate intubation; his entire abdominal viscera were exposed. The patient was taken directly to the operating room for silo placement, which was anchored to his abdominal wall fascia and placed on external traction (Figure 2). In the operating room, the defect was confirmed to be to the right of the umbilical cord insertion and involved the small bowel, colon, entire liver, spleen, and stomach; there was no sternal involvement. Abdominal domain was nonexistent. The fascial defect was extended to the pubis to prevent any compression on the protruding abdominal organs. To create a silo large enough to encompass the entire defect, two large sheets of silastic mesh were sewn to the abdominal fascia with 3-0 polypropylene sutures and felt pledgets (Figures 3(a) and 3(b)). An external traction/pulley system was fashioned to the top of the silastic bag. Antibiotic ointment was applied to the sides of the silo and fascial edges, and the entire silo was wrapped in dry gauze (Figure 4).

#### 2.1.2. Initial Management

**Figure 5:**
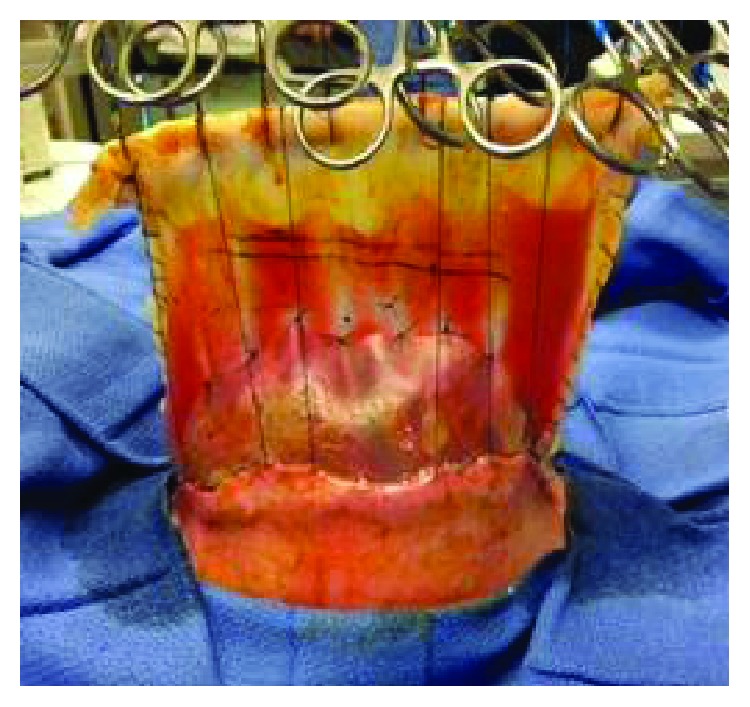
Placement of polypropylene sutures in the mesh to encourage visceral reduction. Polypropylene sutures were placed in the mesh silo to encourage reduction of the viscera into the abdominal cavity.

To manage the massive defect, rows of sutures were placed on the upper part of the silo to encourage descent of the bowel (Figure 5). The defect was tightened once or twice per week, while the silo dressing was changed daily. To maintain fascial traction, the pulley system was readjusted daily to ensure that the patient's lower back was slightly elevated from the bed. Through this technique, we felt that we would be able to encourage growth of all layers of the abdominal wall by using the patient's body weight to provide traction. Once per week, the silo was opened under sterile conditions for several purposes: (1) to release any adhesions of the herniated viscera to the fascial edges, (2) to send cultures for infection surveillance, (3) to wash out the silo with warmed bacitracin solution to help prevent infection, and (4) to add Seprafilm® (Genzyme Corporation, Cambridge, MA) to help minimize adhesion formation. The patient was also maintained on cefazolin for prophylaxis against skin bacteria during the prolonged silo phase of abdominal wall growth.

Due to his significant pulmonary hypoplasia, our patient was transitioned to high-frequency jet ventilation, where he remained for the first six weeks of life. Along with persistent respiratory failure, the patient demonstrated a significant inflammatory/capillary leak response, contributing to diffuse anasarca and renal dysfunction. He lived in a state of acute-on-chronic abdominal compartment syndrome due to a combination of fluid overload and compression of the kidneys during silo tightening. The patient's renal dysfunction improved after less aggressive tightening of the silo and initiation of a combination of diuretics and albumin.

#### 2.1.3. Abdominal Closure

**Figure 6:**
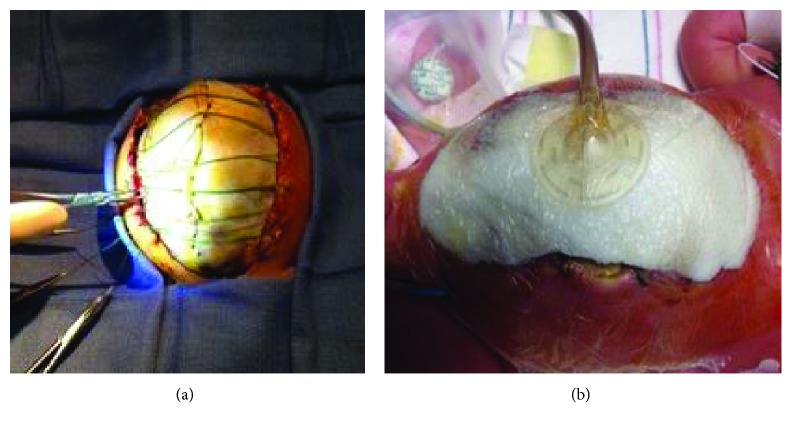
Placement of acellular dermal allograft mesh for covering and use of braided polyester sutures for tension-inducing abdominal wall growth. After placement of the allograft, 4-0 braided polyester sutures were placed on either side of the abdominal fascia using Roeder's knots, thus allowing for intermittent tightening without replacement of the sutures (a). A wound vac sponge was placed on top of the mesh and sutures (b).

**Figure 7:**
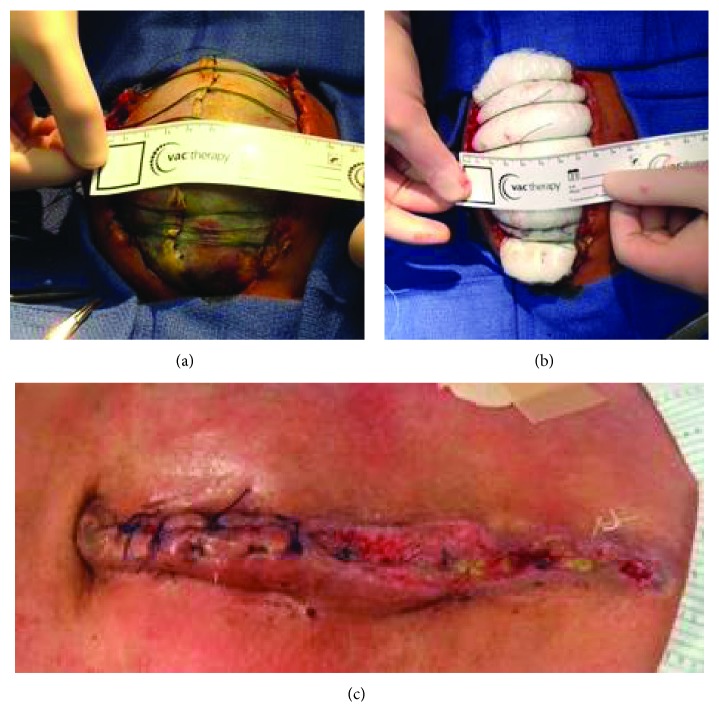
(a–c) Sequential closure of the abdominal fascia. Tightening of the retention sutures was done on a weekly basis until the fascia could be completely closed (a, b). The allogaft mesh was removed upon final closure of the abdomen (c).

At one month of age, the abdominal contents had been reduced to the level of the abdominal fascia; therefore, he was transitioned to covering his intestinal viscera with an acellular dermal allograft (as a fascial bridge) with adjustable tension sutures placed on the fascia to continue abdominal wall growth. The goal was to provide a biologic closure and covering of the abdominal viscera while allowing ongoing growth of the abdominal wall fascia. The allograft (8 × 12 cm) was sewn to the fascia using horizontal mattress sutures, and a white negative pressure sponge encased in a plastic covering with drainage holes was laid on top. Ethibond® (Ethicon, Johnson & Johnson, Belgium) sutures (2-0 in size) were placed in a horizontal mattress fashion with pledgets, connecting the two sides of the abdominal fascia; these sutures were secured with Roeder's knots to allow for intermittent tightening of the fascia without needing to replace the sutures (Figures 6(a) and 6(b)). Wound dressing change with tightening of the fascial sutures was performed 1-2 times weekly until the fascia was able to be closed (Figures 7(a) and 7(c)). The patient was able to transition to the conventional ventilator 17 days after allograft placement.

#### 2.1.4. Intestinal Atresia Repair

At 4.5 months of age, the patient still did not have bowel function. A contrast enema revealed a small-caliber colon with inability to reflux contrast into the small bowel and dilated loops of small bowel. He was taken electively to the operating room, where a type I ileal atresia and Meckel's diverticulum were found and repaired. During this operation, a portion of the allograft was resected, a gastrostomy tube was placed, and a series of adjustable fascial closure sutures were placed over a covered negative pressure dressing to complete his abdominal closure over the next two weeks.

#### 2.1.5. Discharge

From a neurologic perspective, follow-up brain imaging was negative for any intracranial bleeding but did show ventriculomegaly that remained stable after 2 weeks of age. Due to his significant pulmonary hypoplasia, prolonged ventilation requirements, and significant tracheomalacia, a tracheostomy was placed at 3 months of age. He was discharged on mechanical ventilation. Despite full continuity of the GI tract, the patient was initially fairly intolerant of feeds, requiring slow progression with continued support of parenteral nutrition. Due to high bilirubin levels from prolonged parenteral nutrition exposure, Omegaven® (Fresenius Kabi, Auckland, NZ) was started. A GJ tube was also placed prior to discharge for gastric dysmotility. The patient was diagnosed with hypothyroidism and was started on levothyroxine at 2 months of age. Despite undergoing 37 procedures in total and spending 241 days in the ICU, the patient was discharged to a long-term care facility at 9 months of age and discharged home at 16 months on a ventilator. He is currently tolerating full enteral feeds through a gastrostomy tube, is off parenteral nutrition, and is tolerating his ventilator wean program.

### 2.2. Case #2: Prenatal Care and Delivery

**Figure 8:**
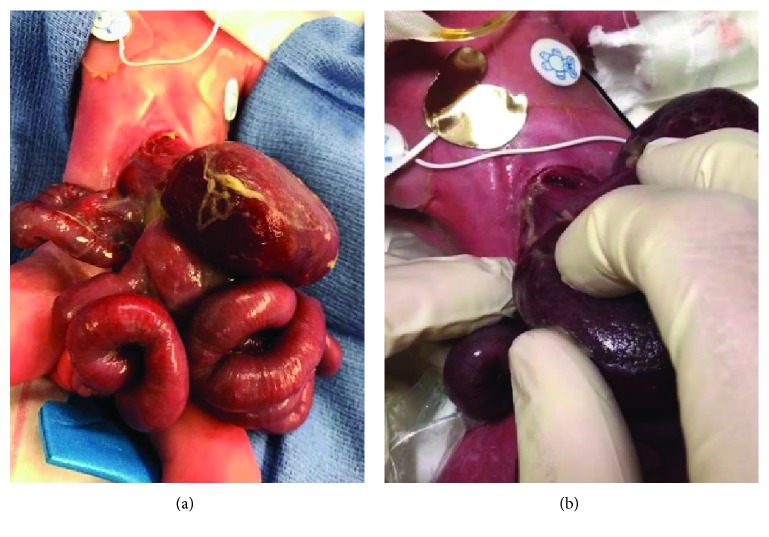
(a, b) Patient #2 at delivery. This figure shows the large abdominal defect with bowel, liver, and stomach herniation of patient #2 at birth before transfer to the operating room (a). The defect was to the left of midline with no identifiable sac. The very apex of the heart can be seen at the cranial edge of the defect (b).

Our second patient is a female infant with a giant gastroschisis and concern for pentalogy of Cantrell. The fetus also was found to have complete chorioamniotic separation with oligohydramnios and intrauterine growth restriction. Due to premature rupture of membranes, the patient was born at 30 6/7-week gestational age with a birth weight of 1.2 kg. APGAR scores at birth were 4, 5, and 8; she was intubated for respiratory insufficiency prior to transfer to the operating room (Figure 8).

#### 2.2.1. Initial Operation

**Figure 9:**
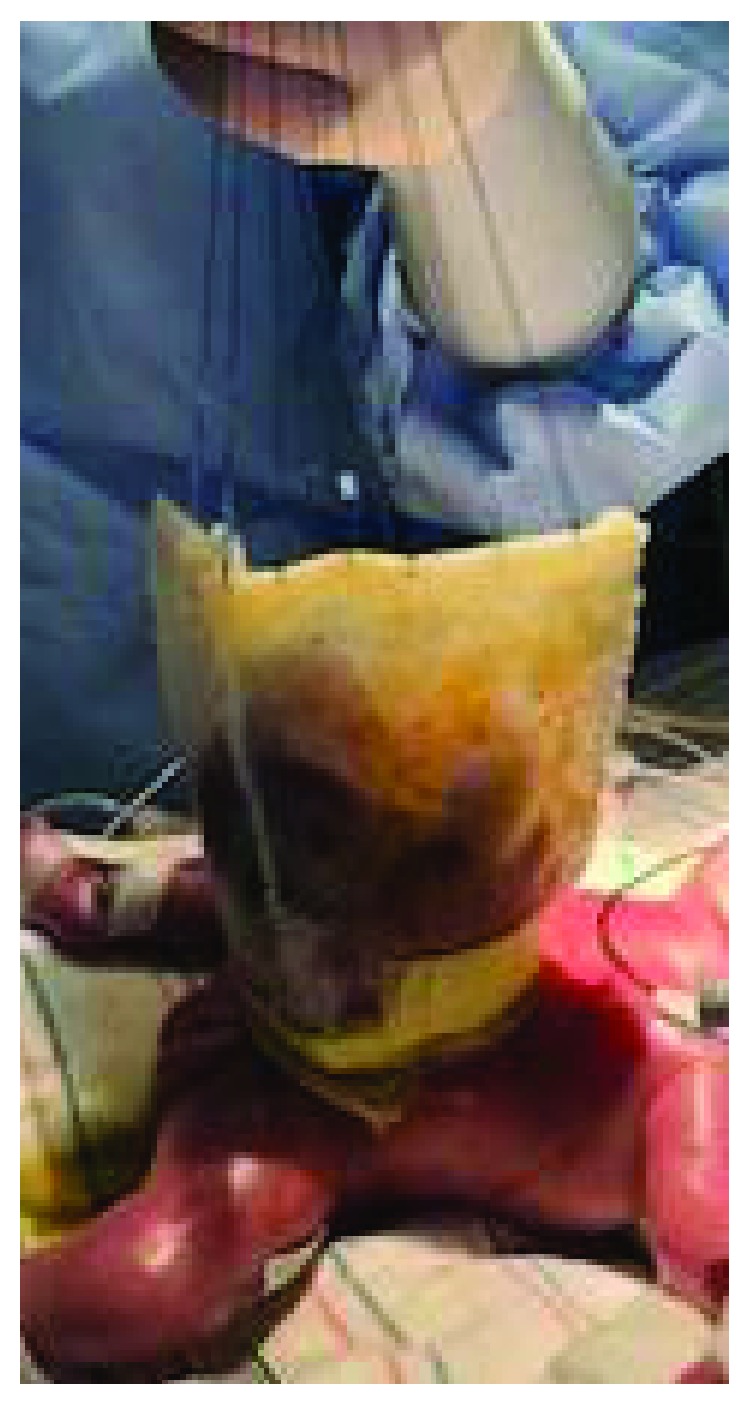
External traction with silo covering for patient #2. To cover the bowel and allow for growth of the abdominal cavity, a silastic sheath was sewn to the abdominal fascia. The silo will be suspended around the isolette for external traction on the abdominal fascia. A dressing that included bacitracin, petrolatum gauze, and dry gauze bandages was applied.

In the operating room, she was found to have a giant gastroschisis defect to the right of the umbilical cord insertion with herniation of the small bowel, colon, entire liver, stomach, spleen, and pancreas. The defect extended to the level of the xiphoid and anterior diaphragm, with exposure of the apex of the heart. There was no membrane to suggest a ruptured omphalocele, and the viscera had the gross appearance of chronic amniotic fluid exposure consistent with gastroschisis. A similar silastic silo was sutured to the fascia. 2-0 polypropylene sutures were used to close the top of the silo and then to suspend the silo for traction (Figure 9). The silo was dressed with antibiotic ointment, surrounded with petrolatum gauze, and gauze bandages.

#### 2.2.2. Initial Management

Her postoperative course was complicated by marked physiologic instability, including pressor requirement (dopamine, epinephrine, and norepinephrine—especially during attempts at diuresis), persistent albumin supplementation, and blood product transfusion due to capillary leak syndrome with diffuse anasarca, oliguric renal failure, proteinuria, and hypoxic respiratory failure requiring both jet ventilation and then high-frequency oscillatory ventilation. She was placed on an aggressive diuretic regimen with chlorothiazide, bumetanide, and 25% albumin. She initially responded to these measures; however, she was found to have nephrotic syndrome and ultimately developed acute kidney injury that progressed to renal failure. Chemical paralysis was initiated during the fifth week of life due to increasing respiratory acidosis and ventilatory settings.

#### 2.2.3. Bridged Abdominal Closure

**Figure 10:**
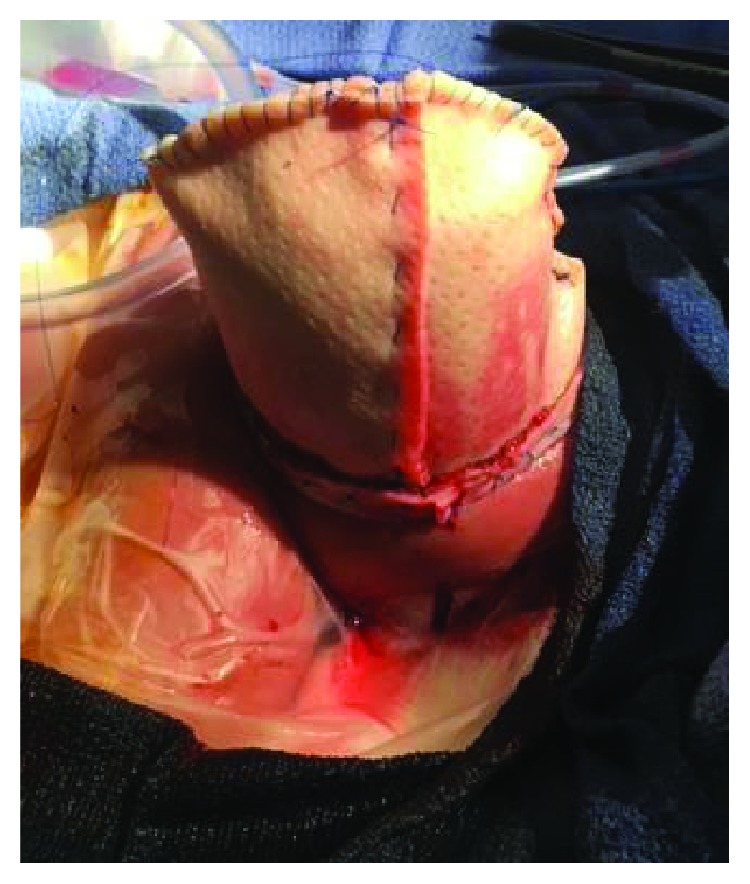
Negative pressure dressing placement in patient #2. Placement of a mesh silo with a negative pressure dressing in patient #2.

Due to the continued fluid losses and a persistent inflammatory reaction, her abdominal silo was changed, and a Strattice™ (LifeCell, Somerville, NJ) mesh was sutured to the fascia using pledgeted 4-0 polypropylene sutures at 33 days of age. 3-0 polypropylene sutures were used to close the opposing edges of the mesh, forming an omphalocele-like mesh covering, with the goal to provide a more biologic covering to decrease fluid losses. A negative pressure dressing was placed on top of the Strattice™ mesh and placed to −125 mmHg of continuous suction (Figure 10).

#### 2.2.4. Final Care

Despite placement of the biologic silo, she continued to require high levels of hemodynamic support and to have progressive renal failure with electrolyte derangement. Due to her persistent multiorgan system failure, the family elected to redirect care, and she died on DOL 38.

## 3. Discussion

In this case report, we described the management complexities of two patients with giant gastroschisis and total liver herniation, survival of which has not been reported until now. These patients were managed using lessons learned from the care of patients with ruptured omphalocele—including the use of external traction to induce growth of the abdominal wall.

While up to 20% of patients with gastroschisis are unable to underdo primary fascial closure due to the size of the defect and the amount of intra-abdominal visceral herniation [8], only two studies have been published describing the management of patients with gastroschisis and liver herniation. Morris et al. described a 36-week gestational male with gastroschisis and herniation of the left hepatic lobe [9]. A 5 cm silo was able to be placed with reduction of the bowel in 5 days; however, liver reduction was incomplete, so a negative pressure wound therapy device was applied. This allowed for abdominal coverage without need of a silo, and the patient's abdomen was fully closed by day 11 [9]. Fetal MRI and postnatal diagnosis only showed 30% liver herniation in this case, allowing for some abdominal pressure to contribute to the formation of the thoracic cavity, compared to our patients with total liver herniation. In a retrospective review by McCllelan et al., 6 out of 117 patients with gastroschisis had liver herniation, with four described as having most or a central lobular portion of the liver herniated [10]. All of these patients required silo placement and were more likely to require a biologic patch for closure; unfortunately, all patients in this study with major liver herniation had pulmonary hypoplasia and ultimately died [10]. The neonate born with giant gastroschisis faces many challenges, including multiple procedures before the abdomen can be closed; pulmonary hypoplasia requiring long-term ventilation (with the use of advanced ventilation techniques, such as high-frequency jet ventilation); the potential for intestinal malabsorption and dysfunction that requires parenteral nutrition; renal dysfunction from fluid shifts, third spacing, and compression during reduction of the abdominal viscera; and prolonged sedation and analgesic requirements.

It is with advancement in critical care medicine that these patients have a chance at survival. Those born prematurely have an increased risk for decompensation that requires even more intensive critical care management. Pacilli et al. concluded that the survival rate of these infants depends upon the degree of visceroabdominal disproportion, along with the presence of associated anomalies and respiratory distress at birth [6]. This could partly explain why patients with giant gastroschisis have a poor prognosis. The ability to establish some of these abnormalities prenatally provides the opportunity for appropriate prenatal counseling, prognosis, and postnatal expectations.

For patients with giant abdominal wall defects, the degree of pulmonary hypoplasia can be measured using fetal imaging. In 2007, Kamata et al. studied the utility of measuring the lung/thorax transverse area ratio (L/T) and the chest/trunk length ratio (C/T) in determining the degree of pulmonary hypoplasia on prenatal ultrasound. A decreased L/T ratio was significantly predictive of pulmonary hypoplasia with poor prognostic factors including a giant omphalocele with a ruptured membrane, pulmonary hypoplasia, and herniation of the majority of the liver [3]. This echoes his initial study from 1996, where 6/7 patients with a ruptured omphalocele died due to respiratory insufficiency from pulmonary hypoplasia [11]. More recently, Danzer and colleagues described the use of fetal MRI to measure total lung volume (TLV) in patients with giant omphaloceles. Patients with <50% predicted TLV required prolonged ventilator support and longer hospitalizations, and two patients required a tracheostomy [4]. For both of our cases, pulmonary hypoplasia presented a significant comorbidity. The patient in case #2 also had premature lungs, requiring surfactant upon birth, which also contributed to her high level of ventilator support.

Another concern with these patients is the development of abdominal compartment syndrome during reduction of the abdominal viscera, which can aggravate respiratory failure and contribute to renal dysfunction. In our patients, baseline renal ultrasound studies were performed not only to look for congenital anomalies but also to look at resistive indices, with increasing values during reduction used as a sign of compression. Renal dysfunction was noted in both patients which at times required loosening of the silo to release compression. Lacey et al. described using bladder pressure monitoring with use of a Foley catheter to guide reduction in patients with abdominal wall defects [12]. In their prospective study of 42 infants with either gastroschisis or omphalocele, 12 patients had altered management in the form of a staged closure versus primary repair based on increased bladder pressure (>20) in the operating room. Similarly, Santos Schmidt and colleagues described using an intraoperative bladder pressure of 20mmH_2_O as a threshold for deciding on a primary closure or delayed reduction in 45 neonates with gastroschisis [13]. Based on this threshold, Santos Schmidt et al. retrospectively found no difference in complications, time to oral feeding, length of hospital stay, or incidence of oliguria, concluding that intraoperative bladder pressure monitoring can help guide surgical decision-making for patients with gastroschisis [13]. In patients with large abdominal wall defects and visceroabdominal disproportion, abdominal compartment syndrome with reduction can lead to renal dysfunction and must be closely monitored. Physicians must be aware of the large quantity of insensible fluid losses that may occur with the abdominal cavity enclosed in a silo. Enlisting the help of nephrology specialists early in the intensive care course is crucial to help minimize peripheral edema. When fluid losses remain high despite replacements, early use of a biologic mesh to enclose the abdomen is another option to minimize insensible fluid losses.

Upon review of the literature on the management of giant omphaloceles, multiple different surgical techniques have been employed to manage the abdominal wall defect. Some of the treatment options include epithelization of the amniotic sac with future ventral hernia repair [5, 14], bridging the defect with either prosthetic or biologic mesh, the use of skin flaps (as Gross described in 1948) [6, 14], the use of tissue expanders [7, 8], or the use of a delayed multilayered flap closure that incorporates both the epithelized omphalocele sac and the peritoneum [15]. Using a different technique, Pacilli et al. described making a silo out of a Prolene mesh and suturing it to the fascia just outside the omphalocele defect. Manual reduction was then performed under sedation, and the abdomen was able to be closed without removal of the amniotic sac in a median of 26 days [6]. Similarly, Patkowski et al. described the use of a prosthetic mesh attached to the defect with a Kirschner wire inserted on top of the mesh to provide external traction [8]. These two design concepts are similar to the external traction system used with our patients, where the silo is sutured to the abdominal fascia, and the traction forces are used to encourage abdominal wall growth. In Patkowski et al.'s study, all patients were paralyzed while on traction; our patients initially required chemical paralytic only around reductions due to initial respiratory compromise, and this was not used continuously in the patient who survived [8]. With patience, use of an external traction system will encourage growth of the fascia and abdominal wall while slowly reducing the gastroschisis until the abdominal viscera are at the level of the skin. At this point, abdominal wall growth was encouraged with transversely placed traction sutures to allow for a permanent fascial closure with or without mesh augmentation. For giant omphalocele cases, surgical attempts at closure are generally delayed 3-6 months after initial epithelization for patients with any additional cardiopulmonary comorbidities. This is not possible for gastroschisis or ruptured omphalocele patients.

## 4. Conclusion

Intensive critical care resuscitation and monitoring are imperative to the survival of infants with giant gastroschisis and total liver herniation. Advanced ventilator techniques and monitoring of renal function are important for survival of these infants after birth, while traction-based abdominal wall growth can allow closure of the abdomen. However, extensive prenatal counseling and discussions about the postnatal course, complications, and risk of death are essential as survival, while possible, is well below that of other gastroschisis patients.
